# Injection of Pulmonary Cavities

**Published:** 1874-11

**Authors:** 


*AMERICAN JOURNAL OF THE MEDICAL SCIENCES.—October:*

INJECTION OF PULMONARY CAVITIES.—*By Dr. Pepper.*—* * *

In concluding our study of this question, the following points appear to have been established:

1. That the idea of opening lung cavities by an incision through the chest-walls is at least as oldas Baglivi (probably much older;) but that, owing to the very imperfect character of early clinical records of thoracic diseases, it is difficult to show that such an operation was actually performed before the last century (Barry,) or more probably the present one (Hastings and Storke.)

2. That the idea of conducting continuous treatment of such cavities by local applications made directly through the chest-walls, has been seriously entertained only within the past few years.

3. That the possibility of puncturing the lung in a state of health, with delicate needles, without injury, was demonstrated in a few instances by the advocates of acupuncture; and more recently, in the lower animals, by Koch and others.

4. That the operations of Storks and Mosler have shown that lung cavities are very tolerant of external interference, and that they may be cut down upon and opened, canulæ introduced and retained, and various medicinal agents injected in solution or spray (Mosler.)

5. That the independent observations reported in full in this paper have shown that the continuous treatment of lung cavities by repeated injections, by means of delicate canulæ, may be conducted without pain, hemorrhage, traumatic irritation, or interference with internal medication and hygienic measures.

6. That the cases which are best adapted for this local treatment are those where a single, superficial and circumscribed non-tuberculous cavity exists; but that even when there is implication of the rest of the lung, or incipient disease of the opposite lung, some benefit may be expected.

7. That the mode in which such local treatment does good, is chiefly by altering the character of morbid action in the walls of the cavity, diminishing the amount of purulent formation, as well as the degree of hectic irritation and the danger of constitutional infection. That a certain amount of rest for the walls of the cavity is secured by the marked relief afforded to the cough. Also that it is indicated, by the progress of the cases above reported, that this treatment may favor the cicitrization and contraction of such cavities.

8. That in the cases in which it has been employed (in which over seventy injections have been given,) it has shown itself free from all danger, and of a *certain degree of positive clinical value*, since, during its use, uniform improvement to an exceptional degree has taken place in both the general and local conditions of the patients.

